# Prognostic Value of Modified Lateral Pillar Classification in Legg-Calvé-Perthes Disease

**DOI:** 10.4055/cios.2009.1.4.222

**Published:** 2009-11-25

**Authors:** Dam Seon Lee, Sung Taek Jung, Ki Hyeong Kim, Jae Joon Lee

**Affiliations:** Department of Orthopedic Surgery, Chonnam National University Medical School & Hospital, Gwangju, Korea.

**Keywords:** Legg-Perthes disease, Lateral pillar classification, Prognosis

## Abstract

**Background:**

To evaluate the usefulness of the modified lateral pillar classification as a prognostic factor in Legg-Calvé-Perthes disease (LCPD).

**Methods:**

Thirty nine patients diagnosed with lateral pillar C in LCPD from May, 1977, to October, 2001 were reviewed, and their skeletal maturity was followed. The mean follow up duration was 12 years and 7 months (4 years, 6 months to 24 years, 9 months). Lateral pillar C classification was divided into C1 (50-75% collapse of the lateral pillar) and C2 (> 75%). All radiological and clinical prognostic factors were evaluated. The final results were evaluated according to the Stulberg classification.

**Results:**

Twenty one and 18 of the affected hips were in groups C1 and C2, respectively. According to the Stulberg classification, the final results of group C1 were better than those of C2 (*p* = 0.002). Patients with more head-at-risk signs had significantly poorer outcomes.

**Conclusions:**

The modified lateral pillar classification has significant value for predicting the prognosis of LCPD.

There are a variety of options for treating Legg-Calvé-Perthes disease (LCPD) ranging from conservative to surgical treatment. The treatment modality is chosen according to the degree of involvement of the femoral head and the judgment of the surgeon. Unfortunately, there are no established prognostic factors for LCPD that may be helpful in the early phases of the disease or in the initial diagnosis, and the reliability of those suggested by other authors are controversial.^1^-5) The treatment procedures usually result in poor outcomes in type C hips classified using the lateral pillar classification.6) This study examined the prognostic factors affecting clinical outcomes of type C hips according to the lateral pillar classification and evaluated their efficacy using a modified lateral pillar classification.

## METHODS

### Materials

Between May 1977 and October 2001, 630 patients were treated for LCPD at our institution. Of these, 39 patients with type C hips, which could be followed up until skeletal maturity, were enrolled in this study. There were 33 males (84.6%) and 6 females (15.4%). The affected side was the right in 19 (48.7%) cases and the left in 20 cases (51.3%). The mean age at the onset of the disease at the last follow-up was 7.3 years (range, 2.3 to 12.1 years) and 20.0 years (range, 13.6 to 28.2 years), respectively. The mean follow-up period ranging from the end of the pathological process to skeletal maturity was 12 years and 7 months (range, 4 years and 6 months to 24 years and 9 months). Based on the plain radiographs of the femoral head taken at the initial diagnosis, 18 (46.2%) hips were in the initial stage, 11 (28.2%) were in the fragmentation stage, and 10 (25.6%) were in the late fragmentation stage. Surgery was performed on 7 of the 18 initial stage patients. Based on the plain radiographs, 1 procedure was performed in the early stage while the other 6 were performed in the later stages. Nine cases were treated conservatively using an aid. When these patients were subdivided into 2 groups according to our modified lateral pillar classification, there were no differences in the use of surgery or aid between the groups. The surgical options were taken after the initial stage of the disease in 17 (43.6%) cases: proximal femoral varus osteotomy in 15 cases, Salter innominate osteotomy in 1, and fusion of the greater trochanteric epiphyseal plate in 1. Conservative treatments using an aid were performed in the remaining 22 (56.4%) cases.

### Methods

**Fig. 1:**
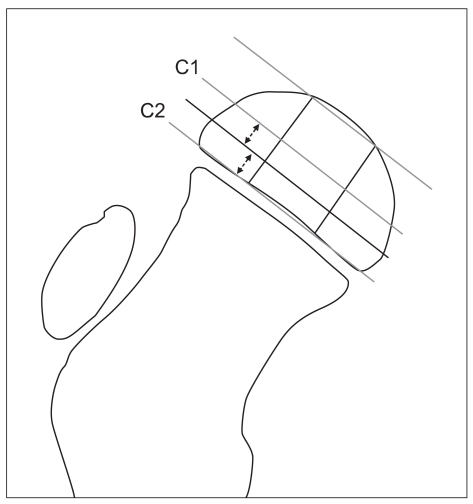
Schematic view of the modified lateral pillar C classification of Legg-Calvé-Perthes disease.

The age at the onset of the disease, gender, and surgical experience were investigated clinically, and the associations between these findings and the final outcomes were assessed according to the Stulberg classification at skeletal maturity. The modified lateral pillar classification system was used to divide the hips into two types, C1 and C2. Type C1 hips were defined as those with 50-75% collapse of the lateral pillar and type C2 hips as those with ≥ 75% collapse of the lateral pillar (Fig. 1). The relationships between the final outcomes and the modified lateral pillar classification system were assessed.

The final outcomes were assessed by evaluating the anteroposterior pelvic radiographs taken at skeletal maturity according to the Stulberg classification system.4) Class I and II hips were graded as good while class III, IV, and V hips were rated as poor. During the follow-up, the head-at-risk signs described by Catterall1) (Gage's sign, calcification lateral to epiphysis, diffuse metaphyseal reaction, lateral subluxation of the femoral head, and horizontal growth plate) were evaluated on the plain pelvic radiographs. Any associations between the number of these radiographic signs and the Stulberg classification were examined.

Statistical analyses were performed using SPSS ver. 12.0 (SPSS Inc., Chicago, IL, USA). A chi-square test was performed to determine if the prognostic factors were associated with the final outcomes assessed according to the Stulberg classification system. A Fisher's exact test was used when an expected value in the crosstabulations was < 5. A *p*-value < 0.05 was considered significant.

## RESULTS

### Correlation between Our Modified Lateral Pillar Classification and the Stulberg Classification

**Fig. 2:**
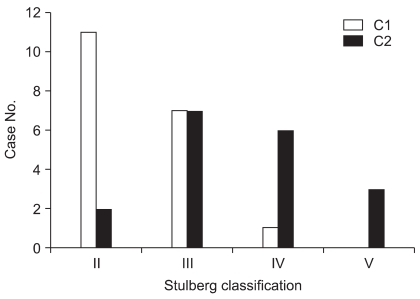
Relation of the modified lateral pillar C classification with the Stulberg classification (chi-square test, *p* = 0.002).

Patients with type C hips according to the Herring classification based on the plain pelvic radiographs taken at the fragmentation stage were included for analysis. The patients were divided into two groups according to the modified lateral pillar classification system. Of the 39 cases, there were 21 (54%) and 18 (46%) cases of C1 and C2 hips, respectively. The age at the onset of the disease, Catterall group, and treatment method were similar in the two groups (*p*-value > 0.05) (Table 1). In the C1 group, 13 (62%) cases had good results (Stulberg I and II) while 8 (38%) cases exhibited poor results (Stulberg III, IV, and V). In the C2 group, 2 (11%) cases had good results (Stulberg I and II) but 16 (89%) cases had poor results (Stulberg III, IV, and V). The prognosis was better in the C1 group than in the C2 group (chi-square test, *p* = 0.002) because the number of patients with poor results (Stulberg III, IV, and V) at the last follow-up radiographic assessment was significantly higher in the C2 group (Fig. 2). More head-at-risk signs were observed in the C2 group than in the C1 group (*p* = 0.014). With regard to the final outcomes excluding the influence of the head-at-risk signs, the C2 group had significantly poorer outcomes than the C1 group (chi-square test, Mantel-Haenszel test, *p* < 0.05).

### Correlation between each Head-at-risk Radiographic Signs and the Stulberg Classification

There was a correlation between the number of Catterall head-at-risk radiographic signs and the Stulberg classification. In particular, the prognosis was worse in those with more head-at-risk signs (Chi-square test, *p* < 0.05). With regard to the correlation between each of the radiographic signs and the final outcomes, Gage's sign, lateral subluxation of the femoral head, and horizontal growth plate except for the remaining two signs were associated with the final outcomes (Table 2).

### Correlation between the Age at the Onset of the Disease and the Stulberg Classification

The patients were divided into those who were < 6 years old and those who were ≥ 6 years old at the age at disease onset. A chi-square test was used to assess the correlation. No association between the age at the onset and the Stulberg classification was found (*p* = 0.336). However, when the patients were divided into 2 groups using 6, 7, 8, and 9 years of age as the dividing point, respectively, those who were < 6 years old at the time of onset had good results (Stulberg I and II) (*p* = 0.039). There was no association observed in the remaining age groups. Other prognostic factors, such as gender and surgical experience, were not related to the Stulberg classification (Table 2).

### Correlation between Number of Head-at-risk Radiographic Signs and the Stulberg Classification

**Fig. 3:**
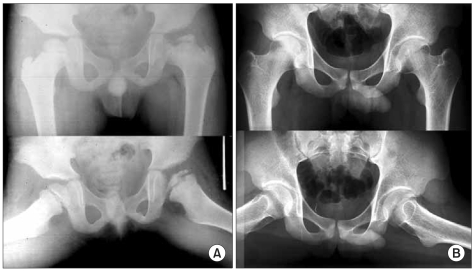
(A) An example of a left hip in group C1 of the modified lateral pillar classification in a patient who was 5.5 years old at presentation. This radiograph shows three radiographical head-at-risk signs, lateral subluxation of femoral head, calcification lateral to epiphysis and diffuse metaphyseal reaction. (B) Anteroposterior and frog-leg lateral radiograph of the pelvis made at the age of eighteen years showing a Stulberg II femoral head with slight differences between the two hips. The patient was treated with a brace.

**Fig. 4:**
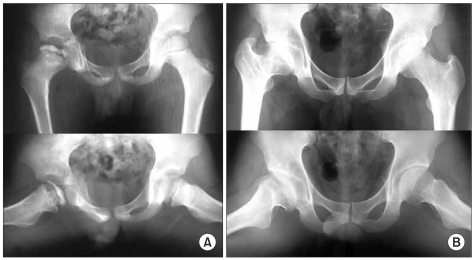
(A) An example of a right hip in group C1 of the modified lateral pillar classification in a patient who was 4.3 years old at presentation. The radiograph shows the four radiographical head-at-risk signs, lateral subluxation of femoral head, Gage's sign, calcification lateral to epiphysis and diffuse metaphyseal reaction. (B) Anteroposterior and frog-leg lateral radiograph of the pelvis, made at the age of sixteen years, showing the Stulberg III femoral head with marked differences between the two hips. The patient was treated with skin traction followed by a cast and with brace one year later.

**Fig. 5:**
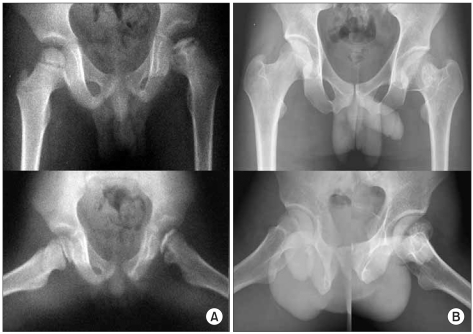
(A) An example of a left hip in group C2 of the modified lateral pillar classification in a patient who was 5.6 years old at presentation. This radiograph shows the two radiographical head-at-risk signs, Gage's sign and a diffuse metaphyseal reaction. (B) Anteroposterior and frog-leg lateral radiograph of the pelvis, made at the age of seventeen years, showing the Stulberg II femoral head with a decreased neck-shaft angle. The patient was treated with a brace.

**Fig. 6:**
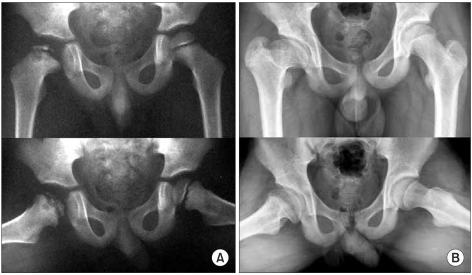
(A) An example of a right hip in group C2 of the modified lateral pillar classification in a patient who was 3.2 years old at presentation. This radiograph shows three significant radiographical head-at-risk signs, lateral subluxation of the femoral head, calcification lateral to the epiphysis, Gage's sign and a diffuse metaphyseal reaction. Among them, only three signs were related to the prognosis. (B) Anteroposterior and frog-leg lateral radiograph of the pelvis, made at the age of fifteen years, showing the Stulberg IV femoral head with a marked difference between the two hips. Affected hip shows a flattening of the femoral head.

The patients were divided into those with ≤ 1 statistically significant sign and those with ≥ 2 statistically significant signs (Figs. 3-6). There were 19 (45%) hips in the former group. Thirteen (68%) of those were classified as Stulberg I and II hips. There were 20 (51%) hips in the latter group with 18 (90%) of them being classified as Stulberg III, IV, and V hips. Therefore, the final outcome was poorer in those with more head-at-risk signs (Stulberg classification) was (*p* = 0.001) (Tables 3 and 4).

## DISCUSSION

According to Stulberg et al.,4) assessments of the treatment methods for LCPD should be conducted at skeletal maturity because a proximal femoral deformity secondary to an epiphyseal plate injury might appear when skeletal growth begins. However, the loss of symptoms and the radiographic signs of the maintenance of the shape of the normal femoral head during the healing period are often misinterpreted by many surgeons as evidence of complete healing, and accordingly regular follow-up examinations tend not to be carried out. Unfortunately, it is impossible to prevent the degenerative changes, which is the ultimate treatment goal of LCPD, when damage to the growth plate of the femoral head is overlooked, leading to secondary changes.

Various clinical and radiological prognostic factors have been suggested by many authors, even though their reliability is controversial. The clinical factors include the age at the onset of the disease, reduced mobility of the hip joint, and obesity. The radiological factors are the extent of femoral head involvement (the Catterall classification and the Salter-Thompson classification), the location of the affected area (lateral pillar classification), and head-at-risk signs. However, the radiological signs do not often appear in the early stages, making them less useful in the initial diagnosis. In addition, they show poor interobserver agreement.

Wiig et al.7) reported that the Stulberg classification, a predictor of the final outcome of LCPD treatment, could be reliable when experienced observers are involved and recommended the clinical use of a simplified version of the classification, in which LCPD patients were divided into 3 groups according to the shape of the femoral head. Herring et al.8) improved the accuracy and reliability of the classification systems using their modified lateral pillar classification system in which an intermediate group termed the B/C border group was added to the Stulberg classification system. However, there has been little study on the prognostic factors of lateral pillar type C hips, and treatments, regardless of the type, have unfavorable outcomes. The authors of this study encountered various treatment outcomes at skeletal maturity in patients with lateral pillar type C hips according to the Stulberg classification and examined prognostic factors affecting the treatment results.

An accurate evaluation of the prognostic factors is essential for choosing a proper treatment modality between various LCPD treatment options. Kamegaya et al.9) divided their study population, who had the same prognostic factors, such as gender, weight, age at the onset of the disease, radiographic head-at-risk signs, into two groups depending on whether they had undergone surgical or a non-surgical treatment. Based on their observation that more spherical femoral heads could be obtained in surgically treated patients, they concluded that the surgical options should be preferred in LCPD patients with a severe deformity. However, Hefti and Clarke10) reported that the decision regarding the LCPD treatment method in many cases was still contingent on the individual experience of the practitioners rather than on scientific evidence. According to their study, surgery usually becomes an option when subluxation of the femoral head occurs or radiographic head-at-risk signs appear in patients with an advanced age or limited joint mobility. In addition, there are no treatment guidelines that can be applied to different age groups and there is little agreement regarding pelvic osteotomy techniques.

The most common classification systems for moderate LCPD are as follows: the Catterall classification,1) which associates the extent of the epiphyseal necrosis and prognosis; the Salter-Thompson classification,11) which is a two-group classification of the extent of involvement of the femoral head according to the extent of the subchondral fracture in the femoral head; and the lateral pillar classification by Herring, which is a three-group classification using the height of the lateral portion of the femoral head for categorization.2) Accordingly, there have also been many studies on LCPD treatment methods based on these classification systems. In some studies, a lateral pillar classification is described as a good indicator of the prognosis of LCPD and has better prognostic efficacy than the Catterall classification.^12^,13) There is no consensus regarding the association between the onset age and the disease. Gent et al.,14) who examined the outcomes of LCPD in less than 6-year-old patients, reported that the prognosis was poorer in those with a more severe collapse of the lateral pillar.

Generally, lateral pillar type C hips result in the worst outcomes. According to Herring et al.,2) study on lateral pillar type C hips, the shape of the femoral heads was mostly aspherical at the final follow-up in patients with type C hips regardless of their age. In particular, Stulberg III and IV hips were observed in 71% of their study population. Aksoy et al.15) reported that a proximal femoral varus osteotomy performed in ≥ 6-year-old patients with type C hips had a poor prognosis at skeletal maturity in 77% of the population. In particular, all patients ≥ 9-years-old showed poor results. However, Gigante et al.,16) who examined the association between the age of the onset of the disease and prognosis, reported that patients with type C hips obtained better results if they were < 6 years old. Therefore, they concluded that the final outcome was better in younger patients due to the more skeletal changes. According to a recent report by Herring et al.,6) the prognosis was poor in patients with type C hips and severe lateral pillar collapse regardless of the patient's age and treatment method. However, they found that surgical treatments produced better results in patients with type B and B/C hips if they were ≥ 8 years of age, whereas no difference was observed between the surgical and non-surgical treatments in patients < 8 years of age. Nevertheless, not all patients with type C hips have poor results and most of our patients obtained fair outcomes. Therefore, this study examined the prognostic factors affecting the final clinical outcomes and found that the subcategorization of type C hips was helpful in determining the prognosis. In this study, final radiological outcomes were significantly good in patients < 6 years of age. Therefore, the age at the onset of the disease is an important prognostic factor.

Catterall highlighted the extent of the deformity of the femoral head as an important prognostic factor. Herring et al. reported that the site of the deformity was a more important prognostic factor in LCPD patients than the extent of the deformity, and the lateral pillar classification was reported to have high interobserver reliability and was helpful for prognostic judgment. Our modified lateral pillar classification was applied to type C hips with a poor prognosis to analyze the final clinical outcomes. Type C2 hips had a poorer prognosis than type C1 hips. In addition, 3 of the head-at-risk signs were found to be associated with the poor results rated according to the Stulberg classification, and there was a correlation between the number of those signs and Stulberg classification. In other words, the prognostic factors significantly associated with the outcome of LCPD treatment included our modified lateral pillar classification, the number of the radiographic head-at-risk signs, Gage sign, lateral subluxation of the fermoral head, and horizontal growth plate. Poor results could be expected in type C2 hips and in those with ≥ 2 radiographic head-at-risk signs.

In conclusion, the number of radiographic head-at-risk signs was associated with the prognosis in type C LCPD patients. Using our modified lateral pillar classification, poorer final outcomes were observed in C2 hips with more severe symptoms. Therefore, subcagetorizing type C hips, which are generally known to have a poor prognosis, will be helpful in predicting the prognosis.

## Figures and Tables

**Table 1:**
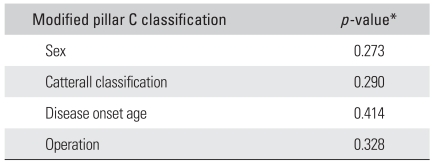
Relationship between the Modified Lateral Pillar C Classification and the Demographic and Radiographic Data.

^*^Chi square test.

**Table 2:**
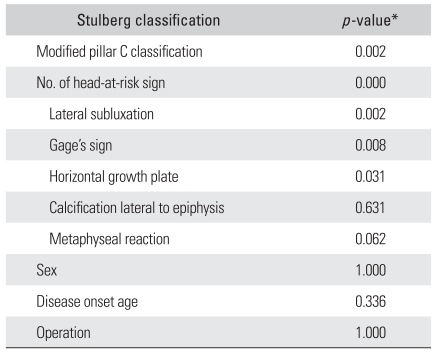
Relationship between Stulberg Classification and the Demographic, Radiographic and Clinical Data.

^*^Chi square test.

**Table 3:**
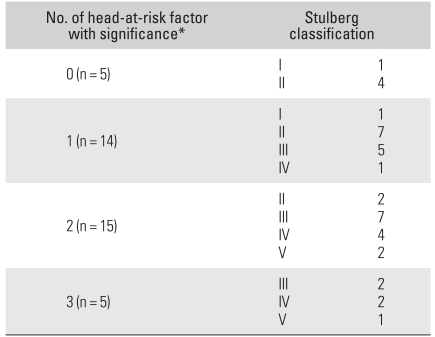
Relationship between the Stulberg Classification and Number of Head-at-Risk Factors with Significance

^*^Head-at-risk factors with significance are lateral subluxation, Gage's sign and horizontal growth plate on radiograph.

**Table 4:**
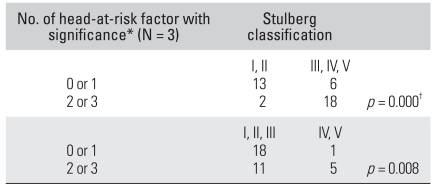
Relationship between the Stulberg Classification with more than 2 Head-at-Risk Factors with Significance

^*^Head-at-risk factors with significance are lateral subluxation, Gage's sign and horizontal growth plate on radiograph, ^†^Chi square test.
